# High frequency of pathogenic non-founder germline mutations in *BRCA1* and *BRCA2* in families with breast and ovarian cancer in a founder population

**DOI:** 10.1186/s13053-018-0094-0

**Published:** 2018-06-05

**Authors:** J. Maksimenko, A. Irmejs, G. Trofimovičs, D. Bērziņa, E. Skuja, G. Purkalne, E. Miklaševičs, J. Gardovskis

**Affiliations:** Institute of Oncology, Riga Stradins University, Dzirciema iela 16, Riga, LV1007 Latvia

**Keywords:** Pathogenic non-founder *BRCA1/2* mutations, Triple-negative breast cancer, Familial breast cancer

## Abstract

**Background:**

Pathogenic *BRCA1* founder mutations (c.4035delA, c.5266dupC) contribute to 3.77% of all consecutive primary breast cancers and 9.9% of all consecutive primary ovarian cancers. Identifying germline pathogenic gene variants in patients with primary breast and ovarian cancer could significantly impact the medical management of patients. The aim of the study was to evaluate the rate of pathogenic mutations in the 26 breast and ovarian cancer susceptibility genes in patients who meet the criteria for *BRCA1/2* testing and to compare the accuracy of different selection criteria for second-line testing in a founder population.

**Methods:**

Fifteen female probands and 1 male proband that met National Comprehensive Cancer Network (NCCN) criteria for *BRCA1/2* testing were included in the study and underwent 26-gene panel testing. Fourteen probands had breast cancer, one proband had ovarian cancer, and one proband had both breast and ovarian cancer. In a 26-gene panel, the following breast and/or ovarian cancer susceptibility genes were included: *ATM, BARD1, BLM, BRCA1, BRCA2, BRIP1, CDH1, CHEK2, EPCAM, FAM175A, MEN1, MLH1, MRE11A, MSH2, MSH6, MUTYH, NBN, PALB2, PMS2, PTEN, RAD50, RAD51C, RAD51D, STK11, TP53,* and *XRCC2*. All patients previously tested negative for *BRCA1* founder mutations.

**Results:**

In 44% (7 out of 16) of tested probands, pathogenic mutations were identified. Six probands carried pathogenic mutations in *BRCA1*, and one proband carried pathogenic mutations in *BRCA2*. In patients, a variant of uncertain significance was found in *BRCA2*, *RAD50, MRE11A* and *CDH1*. The Manchester scoring system showed a high accuracy (87.5%), high sensitivity (85.7%) and high specificity (88.9%) for the prediction of pathogenic non-founder *BRCA1/2* mutations.

**Conclusion:**

A relatively *high incidence of pathogenic non-founder BRCA1/2 mutations* was *observed in a founder population.* The Manchester scoring system predicted the probability of non-founder pathogenic mutations with high accuracy.

## Background

Hereditary breast cancers account for approximately 10% of all breast cancers, and approximately 23% of all ovarian cancers are considered hereditary [1, 2]. According to *Plakhins* et al., *BRCA1* pathogenic founder mutations (c.4035delA, c.5266dupC) contribute to 3.77% of all consecutive primary breast cancers and 9.9% of all consecutive primary ovarian cancers [3]. *BRCA1* and *BRCA2* pathogenic founder mutation analysis is a relatively straightforward and cost-effective screening strategy to identify mutation carriers [4]. In Latvia, all consecutive breast and ovarian cancer cases are eligible for *BRCA1* pathogenic founder mutations (c.181 T > G, c.4035delA, c.5266dupC) screening [5], and the costs of the test are covered by the public health care system. However, according to recent studies, non-founder *BRCA1* and *BRCA2* pathogenic mutations account for up to 21.6% of all *BRCA1* and *BRCA2* pathogenic mutations in the Aschkenazi Jewish population [6, 7]. There is little information about pathogenic *BRCA1/2* non-founder mutations in Latvia. In a study published by *Berzina* et al., pathogenic non-founder mutations in *BRCA1* and *BRCA2* were identified in 4 out of 30 high-risk breast/ovarian cancer families from the Latvian population [8]. In another study published by *Tihomirova* et al., non-founder pathogenic mutations in *BRCA1* and *BRCA2* were detected in 9 out of 160 patients with breast and ovarian cancer [5]. These findings suggest that the proportion of pathogenic *BRCA1/2* non-founder mutations is small and that family cancer history alone is of limited value to find subgroups of individuals, where expensive complete *BRCA1/2* testing is indicated.

The remaining hereditary breast and ovarian cancer cases are associated with mutations in other breast and ovarian cancer susceptibility genes, such as *BRCA1/2*, *TP53*, *PTEN*, *CDH1*, *STK11*, *MLH1*, *MSH2*, *MSH6*, *PMS2*, *PALB2*, *CHEK2*, *ATM*, *RAD51C*, *RAD51D*, *BRIP1* and other [9]. Patients and their relatives harbouring mutations in hereditary cancer predisposing genes could benefit prevention and screening strategies or novel therapeutic approaches [10, 11]. Advances in next-generation sequencing allowed the implementation of low-cost multi-gene panel testing in clinical practice to detect pathogenic mutations in hereditary cancer predisposing genes [12].

Therefore, knowledge of the frequency and phenotypical features of pathogenic mutations beyond *BRCA1* pathogenic founder mutations in *breast* and *ovarian cancer* susceptibility genes is essential for determining the role of second-line testing with multi-gene panels in counselling unsolved *high-risk breast and ovarian cancer patients.*

The aim of the study was to evaluate the rate of pathogenic mutations in the 26 breast and ovarian cancer susceptibility genes in patients who meet the criteria for *BRCA1/2* testing and to compare the accuracy of different selection criteria for second-line testing in a founder population.

## Methods

### Patient group

Sixteen sequential patients with primary breast and/or ovarian cancer who met all inclusion criteria were included in the study between October 2016 and August 2017. The inclusion criteria were as follows: 1) fulfil at least one of the National Comprehensive Cancer network (NCCN) *BRCA1/2* testing criteria (Table 1) (www.nccn.org); 2) previously *tested negative* for *BRCA1* pathogenic founder mutations (c.181 T > G, c.4035delA, c.5266dupC); 3) *able to cover* the *cost* of the 26 multi-gene *tests.*

**Table 1:** NCCN selection criteria for screening of mutations in *BRCA1*and *BRCA*2

At least one of the following criteria has to be met:	
1. Personal history of breast cancer diagnosed < age 45 years	
2. Personal history of breast cancer diagnosed < age 50 years and at least one case of breast cancer at any age in close blood relative	
3. Personal history of triple negative breast cancer diagnosed < age 60 years	
4. Personal history of breast cancer diagnosed at any age and at least two cases of breast cancer diagnosed at any age or at least one close blood relative with breast cancer diagnosed ≤50 years or at least one blood relative with ovarian carcinoma or a close male blood relative with breast cancer	
5. Personal history of ovarian cancer	
6. Personal history of male breast cancer	

The following clinical information was obtained: age at testing, personal cancer history, age at cancer diagnosis, breast and/or ovarian cancer pathology, *BRCA1/2* testing history, a family cancer history that covers a 3-generation pedigree according to probands information. The median patient age was 45.6 years (33–63 years). Fifteen out of 16 (93.75%) patients were females, and 1 out of 16 (6.25%) patients was male. Thirteen patients had unilateral breast cancer, 1 patient had bilateral breast cancer, 1 patient had ovarian cancer, and in 1 patient had both breast and ovarian cancer. Four out of 16 (25%) breast cancers were luminal-like HER2 negative, 2 out of 16 (12.5%) breast cancers were luminal B HER2 positive, 8 out of 16 (50%) breast cancers were triple-negative, and 1 out of 16 (6.25%) breast cancers was HER2 positive. The patient characteristics are summarized in Table 2.

**Table 2:** The baseline characteristics of patient group

Nr.	Probands age at diagnosis (years)	Primary cancer site	Morphological subtype	Breast cancersubtype	Tumor grade	Family history
1	54	Breast	Ductal	Luminal	missing	Mother and maternal aunt – breast cancer age 60; daughter - polycytemia vera age15, brother – melanoma age 60
2	40	Breast	Ductal	Triple-negative*	G3	Mother - Breast and ovarian cancer age 40
3	33 and 38	Left Breast/ Right Breast	Ductal/ Ductal	Triple-negative/ Luminal	G3/G3	Paternal grandmother - unknown primary gynecological cancer age 50
4	63	Breast and Ovaries	Ductal	Triple-negative	G2	Mother with breast cancer age 55; sister - ovarian cancer age 59
5	37	Ovaries	NA	NA	NA	Mother - breast cancer age 64
6	58	Breast	Lobular	Luminal	G2	Mother and maternal aunt – breast cancer age > 60
7	43	Breast	Ductal	Triple-negative	G3	No
8	42	Breast	Ductal/Medullary	Triple-negative	G2	Mother - breast cancer age 60
9	50	Breast	Ductal	Triple-negative	G3	Mother - breast cancer age 52
10	35	Breast	Ductal	Triple-negative	G3	Mother - breast cancer age 46
11	52	Breast	Ductal	Luminal B HER2 positive	G2	Mother and maternal aunt – breast cancer age > 50
12	41	Breast	Ductal	HER2 positive	G3	No
13	53	Breast	Ductal	Triple-negative	missing	No
14	36	Breast	Ductal	Luminal	missing	No
15	53	Breast	Ductal	Luminal	missing	Mother and maternal grandmother – breast cancer age > 60 years
16	40	Breast	Ductal	Luminal B HER2 positive	missing	No

#### DNA testing

Informed consent for genetic testing was obtained for all patients. All patients underwent DNA testing with a 26-gene panel (myBRCA HiRisk Hereditary Breast and Ovarian Cancer screening Test, VeritasGenetics, USA) that is a targeted next-generation sequencing assay for the detection of mutations in 26 breast and ovarian cancer susceptibility genes. The genes included high-penetrance breast-ovarian genes (*BRCA1, BRCA2, PTEN, TP53, CDH1, STK11, PALB2*), moderate-penetrance breast and/or ovarian genes (*CHEK2, BRIP1, ATM*), and additional genes (*BARD1, BLM, EPCAM, RAD50, RAD51C, RAD51D, MEN1, MRE11A, MUTYH MSH2, MLH1, NBN, MSH6, PMS2, FAM175A, XRCC2)*. In all patients, the test was performed using saliva. The specificity and sensitivity of the assay are 99.9% for point mutations and small insertions/deletions in the 24 sequenced genes and 99.9% for structural variations in *BRCA1* and *BRCA2*.

#### Statistical analysis

The specificity, sensitivity and accuracy of the NCCN criteria, Manchester scoring system and Swedish Breast cancer group criteria for the prediction of pathogenic non-founder mutations were evaluated. The Manchester score of 15 points threshold was used to assess the likelihood of *BRCA1/2* pathogenic mutation [13]*.* The specificity, sensitivity and accuracy of different selection criteria for *BRCA1/2* testing in our cohort were calculated using *MedCalc* Statistical Software version 17.9.

## Results

In seven out of sixteen (44%) patients included, pathogenic non-founder *BRCA1/2* mutations were identified. Six patients carried pathogenic variants of *BRCA1* and one of *BRCA2*. In four patients, variants of uncertain significance of *BRCA2*, *RAD50, MRE11A* and *CDH1* were found. Detailed results are shown in Table 3. The NCCN criteria showed a high sensitivity (100%) with low specificity (50%) for the prediction of non-founder pathogenic *BRCA1/2* mutations. The Swedish Breast cancer group criteria showed a low sensitivity (57.1%) with three false negative results. The Manchester scoring system showed a high accuracy (87.5%) for the prediction of pathogenic non-founder *BRCA1/2* mutations with high sensitivity (85.7%) and specificity (88.9%). The sensitivity, specificity and accuracy of different criteria/scoring systems for the detection of probability of *BRCA1/2* pathogenic mutations in our cohort are compared in Table 4.

**Table 3:** Results

Nr.	Mutation	Clinical significance of mutation	NCCN inclusion criteria	Manchester score [13]	Swedish Breast cancer group criteria for screening of mutation in *BRCA1* and *BRCA2*
1	RAD50c.980G > A	VUS	NCCN4	17	One case of male breast cancer
2	BRCA1c.5075-?_5152 +?del	PAT	NCCN2	29	One case of triple-negative breast cancer ≤age 40
3	BRCA1c.1-?_c.134 +?del	PAT	NCCN3	20	One case of breast cancer ≤age 35
4	BRCA2c.6998dupT	PAT	NCCN4	19	Breast cancer and ovarian cancer in one individual.
5	BRCA1c.5117G > A	PAT	NCCN5	15	Do not match
6	RAD50c.251 T > A	VUS	NCCN4		
	MRE11Ac.1715G > A	VUS		6	NA
7	BRCA1c.1961delA	PAT	NCCN3	14	Do not match
8	BRCA2c.280C > T	VUS	NCCN4	14	Do not match
9	BRCA1c.5117G > A	PAT	NCCN4	16	Do not match
10	BRCA1c.4996_4997dupTA	PAT	NCCN4	20	One case of triple-negative breast cancer ≤age 40
11	Negative	Negative	NCCN4	2	Do not match
12	Negative	Negative	NCCN1	2	Do not match
13	Negative	Negative	NCCN3	8	Do not match
14	Negative	Negative	NCCN1	8	Do not match
15	CDH1 c.808 T > G	VUS	NCCN4	8	Do not match
16	Negative	Negative	NCCN1	0	Do not match

*PAT*, pathological; *VUS*, variant of uncertain significance; *Triple-negative breast cancer was defined as ER-0%; PR-0%; HER2- negative;

**Table 4:** Comparison of different selection criteria for *BRCA1/2* testing in our cohort

Criteria	Sensitivity	Specificity	Accuracy
NCCN	100%	50%	64%
Manchester scoring system	85.7%	88.9%	87.5%
Swedish Breast cancer group	57.1%	88.9%	75%

## Discussion

Our study is the first report on the use of a 26 *gene panel* in to examine breast and ovarian cancer susceptibility genes in patients in Latvia. We demonstrated a high frequency of pathogenic non-founder germline mutations in *BRCA1* and *BRCA2* genes. In seven out of sixteen (44%) primary breast and ovarian cancer patients matching the criteria for *BRCA1/2* testing pathogenic non-founder *BRCA1/2* mutations were identified. All 7 pathogenic mutations, including 2 large deletions, are novel in populations of Latvia [5, 8]. These results may suggest that the present practice of testing only the 3 most frequent *BRCA1* pathogenic founder mutations is insufficient and fails to detect a considerable number of pathogenic mutations in *BRCA1/2*. However, our study comprises a relatively small cohort of selected patients. In a study published by *Frank* et al.*,* 21.6% of patients with Ashkenazi ancestry pathogenic non-founder *BRCA1* and *BRCA2* mutations were identified [6]. In contrast, in the Finnish population of high-risk individuals tested negative for 28 *BRCA1/2* pathogenic founder mutations, additional pathogenic mutations in *BRCA1* and *BRCA2* accounted for just 1.2% [12]. Much larger numbers are necessary to assess the real proportion of pathogenic non-founder mutations in the population of Latvia.

Despite the drawbacks of such a small study group, the initial results raised some observations.

Interestingly, probands that carried a pathogenic non-founder mutation had some common features. All six breast cancer patients in our study with proven pathogenic non-founder *BRCA1/*2 mutations had a triple-negative phenotype. It is well established that approximately 80% of all *BRCA1/2*– related tumours have a triple-negative phenotype [14–18]. The prevalence of pathogenic germline *BRCA1/2* mutations in the selected triple-negative breast cancer patients ranged from 9.2 to 34.4% [19–22]. Additional analyses of cDNA microarray data from van’t Veer showed that *BRCA1-*related tumours have a sporadic basal-like breast cancer gene expression profile [23]. Additionally, according to *Richardson* et al., loss of BRCA1 function could play a role in the development of basal-like breast cancers [24]. *Couch* et al. identified *BRCA1/2* pathogenic mutations in 11.2% of triple-negative breast cancer patients and other breast-ovarian cancer predisposing gene mutations in 3.7% of triple-negative breast cancer patients [25].

In our study we used the NCCN criteria for screening pathogenic mutations in *BRCA1* and *BRCA*2, where triple-negative breast cancer is used as a criterion together with an age limit < 60. Only one out of six breast cancer patients in our study who carried a pathogenic *BRCA1/2* non-founder mutation was older than 60 years of age, but in this case, family cancer history was positive in the study published by *Couch* et al.*,* 3.1% of triple-negative breast cancer patients older than 60 years and only 1.4% with no family history of breast or ovarian cancer were diagnosed with *BRCA1/2* pathogenic mutation [25]. Therefore, our study results support the current NCCN guidelines for screening all triple-negative breast cancer patients younger than 60 years of age.

In contrast, the application of the upper age limit for triple-negative breast cancer patients of 40 years (Swedish Breast cancer group criteria for screening for mutations in *BRCA1* and *BRCA2*) would miss several *BRCA*-positive cases in our cohort [26].

Our small study showed the high accuracy of the Manchester scoring system for the prediction of pathogenic non-founder *BRCA1/2* mutations in founder mutation-negative patients. Our finding is supported by several other studies performed on the validation of the Manchester scoring system in populations of UK, Germany and South East Asia [13, 27, 28]. However, larger numbers of cases are needed for comprehensive *validation* of these criteria in the population of Latvia.

Additionally, three out of eight patients tested negative for 26 breast and ovarian cancer susceptibility genes were HER2 positive. According to a recently published study*,* only 9% of *BRCA1*-related breast tumours and 13% of *BRCA2*-related breast tumours were HER2 positive [29]. HER2 positivity is also included in the Manchester scoring system as a *BRCA1/2* probability decreasing factor [13].

Ovarian cancer in a personal or family history was documented in three out of seven patients who carried a pathogenic *BRCA1/2* non-founder mutation. Additionally, in one case, unknown gynaecological cancer was reported in a paternal aunt. According to recent studies, the presence of ovarian cancer in personal or family history of pathogenic *BRCA1* founder-negative breast cancer patients increases the possibility of carrying previously undetected pathogenic *BRCA1/2* non-founder mutations [30, 31]. Recently, in a study published by *Couch* et al., ovarian cancer in family history was documented only in 1 of 54 pathogenic non-*BRCA1/2* mutation carriers with triple-negative breast cancer [25].

In our study, no pathogenic mutations were detected in another 24 genes included in the panel. Some previously published studies demonstrated that the rate of pathogenic mutations in non-*BRCA1/2* genes ranged from 2.9 to 9.3% [32–35].

Four of the 16 (25%) patients were identified to have a variant of unknown significance (VUS) in *BRCA2*, *RAD50, CDH1* and *MRE11.* Unfortunately, due to an insufficient sample size in our study, we cannot elaborate upon those results.

## Conclusion

*A relatively high incidence of pathogenic non-founder BRCA1/2 mutations* was *observed among patients with triple-negative familial breast cancer in a founder population.* The Manchester scoring system predicted the probability of non-founder pathogenic mutations with high accuracy.

## Abbreviations

*ATM*: Ataxia-telangiectasia mutated
*BARD1*: *BRCA1* (Breast Cancer 1) Associated RING Domain 1 gene
*BLM*: Bloom**’**s syndrome gene
*BRCA1*: *Breast cancer susceptibility gene 1*
*BRCA2*: *Breast cancer susceptibility gene 2*
*BRIP1*: BRCA1-interacting protein 1 gene
*CDH*: Cadherin-1 gene
cDNA: Complementary Deoxyribonucleic Acid
*CHEK2*: Checkpoint kinase 2 gene
DNA: Deoxyribonucleic Acid
*EPCAM*: epithelial cell adhesion molecule gene
ER: Estrogen receptor
*FAM175A*: Family with sequence similarity 175A gene
*G2*: *Moderately differentiated*
G3: Well differentiated
HER2: *Human epidermal growth factor* receptor 2
*MEN1*: multiple endocrine neoplasia type 1
*MLH*: MutL homolog 1 gene
*MRE11A*: MRE11 meiotic recombination 11 homolog A gene
*MSH6*: MutL homolog *6 gene*
*MUTYH*: MutY DNA glycosylase
NA: Not applicable
*NBN*: Nibrin gene
NCCN: National Comprehensive Cancer Network
*PALB2*: Partner and localizer of BRCA2 gene
PAT: Pathological
*PMS2*: postmeiotic segregation increased 2
PR: Progesterone receptor
*PTEN*: Phosphatase and tensin homolog gene
*RAD50*: Human homolog of S. cerevisiae *RAD50* gene
*RAD51*: RAD51 paralog D
*RAD51C*: RAD51 homolog C
*STK11*: serine/threonine kinase 11 gene
*TP53*: tumor protein p53 gene
USA: *United States of America*
VUS: Variant of uncertain significance
*XRCC2*: X-ray repair cross-complementing protein 2
